# “Trust, Responsibility, and Making a Difference”: Qualitative Insights into Why First-Line Managers in Healthcare Remain in Their Role

**DOI:** 10.1177/00469580251368724

**Published:** 2025-08-21

**Authors:** Jonas Svanström, Magnus Lindberg, Bernice Skytt, Maria Lindberg

**Affiliations:** 1 University of Gävle, Sweden; 2 Uppsala University, Sweden; 3 Centre for Research and Development Region Gavleborg/Uppsala University, Gävle, Sweden

**Keywords:** first-line managers, healthcare organization, employee retention, employee autonomy, qualitative research

## Abstract

Healthcare organizations face increasing challenges in retaining first-line managers, who play a key role in operational stability and patient care. Studies indicate that approximately half of all first-line managers intend to leave their position within 5 years. This highlights the need for updated knowledge about conditions under which they work. A qualitative descriptive design was used to explore first-line managers’ perceptions of what is important for them to remain in the managerial role. Data were collected through 20 individual semi-structured interviews with first-line managers from a medium-sized healthcare organization in Sweden between March and June 2022. The data were analyzed using qualitative content analysis. The results are presented through the overarching theme *Empowered through support, responsibility, and meaningful contribution*. This theme describes how first-line managers sustain their motivation and commitment by developing confidence in their ability to lead, influence others, and create meaningful change. Their confidence is grounded in support, responsibility, and role clarity. The theme also shows how first-line managers handle challenges, make decisions, and act to improve staff well-being and patient care. Through these actions, they contribute to the development of their units. Trust from their manager, autonomy in decision-making, and support from colleagues enhance first-line managers’ sense of purpose and motivation, encouraging them to remain in their role. Moreover, balanced workloads and opportunities to make a meaningful impact strengthen their commitment. By recognizing and addressing these aspects, healthcare organizations can better support long-term engagement of first-line managers, fostering sustainable leadership and stability.

**Highlights**

- Trust, autonomy, and peer support boost manager retention.
- Boundary-setting and experience sustain role commitment.
- Balanced workloads enable long-term leadership engagemet.
- Meaningful impact on staff and patients drives motivation.

## Introduction

Healthcare organizations are currently facing substantial challenges in retaining first-line managers, who play a crucial role in maintaining operational efficiency and ensuring high-quality patient care. A recent study by Warden et al^1^ highlighted a concerning trend, more than half of all first-line managers intend to leave their positions within the next 5 years. Moreover, a longitudinal Swedish study by Skagert et al^2^ showed that 40% of healthcare managers with staff and budget responsibility left their roles over a 4-year period, emphasizing that actual turnover is a pressing concern—not just a theoretical risk. Such turnover has severe implications for organizational stability, employee morale, and patient care outcomes. For instance, Warshawsky et al^3^ found that high turnover among first-line managers often results in reduced employee well-being, increased patient injuries, and added financial burdens for organizations. Understanding what motivates first-line managers to stay in their roles is critical to addressing this issue. The sustainability of healthcare organizations depends on insights into the organizational, role-related, and individual conditions influencing first-line managers’ decisions to remain in their positions.

The role of a first-line manager is primarily concerned with ensuring the efficient and effective operation of a healthcare unit. This includes overseeing personnel management, fostering a healthy and supportive work environment, managing finances, and ensuring high-quality patient care. At the same time, the manager must also supervise the daily operations of the unit.^4,5^ The literature consistently highlights that this responsibility necessitates balancing demands and expectations from patients, upper managers, and central support service professionals.^6^ A recent scoping review by Edwin et al^7^ revealed that first-line managers often encounter several organizational challenges. These include limited influence in decision-making processes, extensive administrative duties, budgetary constraints, time constraints, and persistent staffing shortages. Managing such competing priorities, along with associated work stress, plays a crucial role in determining work satisfaction and the work situation for first-line managers.^8-14^ These challenges complicate healthcare management and require diverse approaches to meet operational demands.^6^

Research emphasizes the importance of advantageous working conditions for the well-being and retention of first-line managers.^10,11,15^ For instance, Hewko et al^16^ emphasized the importance of work-life balance, sufficient support from immediate managers, and empowerment as important aspects in influencing job satisfaction and retention among first-line managers. The responsibility of upper management to ensure a supportive work environment for first-line managers has been clearly documented.^17^ Moreover, Lundin et al^18^ recently underscored the importance of fostering positive and supportive relationships between first-line managers and their employees, which is fundamental for creating a foundation for mutual trust and constructive support. Perceived support from nursing staff team managed by the first-line nurse manager has been shown to strongly predict job satisfaction among first-line managers, while support from managers is particularly important in reducing turnover intention.^17^ In addition, Svanström et al^19^ found that high levels of support from both managers and colleagues were consistently associated with increased job satisfaction. Additionally, first-line managers have recognized the importance of supporting their fellow managers during heavy workloads and ensuring collective well-being, as mutual support among peers can be a vital factor in retention.^18^

Much of the existing knowledge about what influences first-line managers to remain in their roles is rather dated and may no longer fully reflect the realities of today’s rapidly changing healthcare context. As healthcare systems continue to evolve, with increasing complexity, staffing shortages, and shifting demands, there is a growing need for updated insights into what motivates managers to stay. From a sustainable work-life perspective, understanding what first-line managers themselves consider important for remaining in their role is essential. Such insights are not only relevant for addressing retention challenges, but also for supporting their professional development and well-being. Therefore, the aim of this study was to explore first-line managers’ perceptions of what is important for them to remain in the managerial role.

## Method

### Design

A qualitative descriptive design was used, without the application of a predefined theoretical framework. Consistent with the methodological approach described by Graneheim et al^20^ and Lindgren et al,^21^ the analysis was grounded in the participants’ own descriptions, allowing patterns to emerge inductively from the data. The Standards for Reporting Qualitative Research (SRQR) were used as a checklist in the preparation of this paper,^22^ and are available as a Supplemental File.

### Setting and Sample

This original study forms part of a doctoral project comprising 4 interconnected sub-studies—2 qualitative and 2 quantitative— which received approval through a unified ethical review process. In this study, we used a purposive sample of first-line managers from a medium-sized healthcare organization in Sweden. The only inclusion criterion was that participants had held a first-line managerial role in healthcare for at least 2 years. The objective was to include participants from various healthcare areas and with different numbers of subordinates. This led to the recruitment of participants from primary healthcare, prehospital care, and inpatient care. The first-line managers were divided into groups of those with fewer than 30 subordinates, those with 31 to 40 subordinates, and those with 41 or more subordinates. Out of 158 first-line managers who were eligible, 45 were invited to participate in the study. The invitees were evenly distributed across the aforementioned number of subordinates groups. A reminder phone call was made if no response had been received after 2 weeks. Thirteen declined to participate without giving a reason or citing lack of time, and 12 did not respond. Ultimately, 20 first-line managers, 17 women and 3 men aged between 38 and 64 years (mean 53.5 years), agreed to participate and were included in the study. Their total experience as managers ranged between 2.5 and 16 years (median 8.8 years, mean 7 years).

### Data Collection

The first author with limited research experience collected data through individual semi-structured interviews conducted in March-June 2022 via Zoom, with the first-line managers either in their workplace (n = 14) or at home (n = 6). Before conducting the interviews, written information was sent to the participants, and their written consent to participate in the study was obtained. To get ready for data collection, the first author conducted 2 pilot interviews with managers who did not meet the inclusion criteria of having held a managerial position for at least 2 years. These pilot interviews were used to evaluate the interview guide and practice interview techniques, and they did not result in any changes to the guide. To strengthen methodological competence prior to the analysis, the first author also completed a doctoral course led by the originator of the qualitative method applied in this study. After the first formal interview, the first author consulted with the third and fourth authors, who have considerable experience in qualitative research, to receive feedback and guidance to develop the interview skills before conducting the remaining interviews, and to ensure that nothing essential was missed. The interviewer had no prior relationship with the participants. An open-ended interview guide was used, and the length of the interviews ranged from 49 to 86 min with a mean of 64 min. The interview guide included questions designed to explore what influenced the participants’ decisions to remain in their roles as first-line managers. For example, participants were asked: “What are important for you in your managerial role in order to continue your work?” and “What motivates you to continue your role as a first-line manager?” Additionally, questions encouraged reflection on, for example, personal, organizational, and managerial aspects that could impact their decision to stay in the role. All interviews were digitally audio-recorded and transcribed verbatim into Word files by the first author, with the files stored on a password-protected server.

### Data Analysis

The manifest and latent content analysis of the transcribed interviews was inspired by Graneheim et al.^20^ The transcripts were carefully read and re-read to gain a deeper understanding of the content. Next, the words, sentences, and paragraphs that were in line with the aim of the study were identified as meaning units. The meaning units were then condensed to create a description of what the participants talked about, and each unit was given a code that briefly described its content. Throughout the entire analytic process, the first and last authors collaborated closely. Initial coding was carried out by the first author, followed by regular discussions with the last author to review, refine, and validate the codes. Discrepancies or uncertainties in interpretation were discussed until consensus was reached. The codes were then analyzed for patterns and grouped by similarities, leading to the development of categories. These categories were further analyzed and refined, which led to the formulation of an overarching theme that captured the underlying meaning—the red thread—that ran through the data. All transcripts were re-read, and excerpts were chosen and presented to strengthen and illustrate the findings. This process enhanced trustworthiness and credibility in the analysis. In line with this approach described by Graneheim and Lundman^20,23^, Lindgren et al^21^ and Elo and Kyngäs,^24^ we did not apply the concept of data saturation. Instead, our focus was on achieving sufficient variation and depth in the data to allow for a rich and meaningful interpretation of the phenomenon under study. This was assessed through the recurrence of patterns across participants, and the emergence of detailed and varied descriptions of their experiences. Toward the later interviews, no substantially new insights emerged, which indicated that the data provided a comprehensive and nuanced understanding of the studied phenomenon.

### Ethical Considerations

This study was conducted in accordance with the Declaration of Helsinki and received ethical approval from the Swedish Ethical Review Authority, with registration number 2019-04583. Prospective participants received written information about the study via email. Before the interviews began, the first author provided the participants with written background information and study particulars, including the aim of the study. The information was also repeated verbally, with any questions encouraged and answered. The participants were informed that participation was voluntary, that they could withdraw from the study at any time without explanation, and that confidentiality was guaranteed.

## Results

### Empowered Through Support, Responsibility, and Meaningful Contribution

The results are presented through the overarching theme *Empowered through support, responsibility, and meaningful contribution* which captures how first-line managers sustain their motivation and commitment by developing confidence in their ability to lead, influence others, and create meaningful change. This confidence is grounded in supportive relationships, a strong sense of responsibility, and clarity about their role and purpose. The theme reflects how first-line managers actively navigate challenges, make independent decisions, and engage in purposeful efforts to improve staff well-being and patient care, contributing to ongoing development within their units. The theme is further illustrated through 3 categories described in more detail below, each supported by quotes from the participants to illustrate their experiences and perspectives.

### To Experience Trust, Make Own Decisions and Having Supportive Colleagues

First-line managers described the importance of experiencing trust, having autonomy to make their own decisions and having supportive colleagues in their daily work to continue in the role as a first-line manager. This engendered a sense of faith and increased their self-confidence in their role. The importance of receiving assistance and encouragement from their employees, from first-line manager colleagues in the management team, and from their closest managers was described as crucial to effectively perform managerial tasks. A well-coordinated and collaborative management team was considered essential in creating a work environment from which first-line managers could draw strength and energy. In particular, the first-line managers underlined the importance of receiving support from their manager to alleviate their workload and provide guidance when needed. The first-line managers felt appreciated when their colleagues and managers demonstrated mutual respect and trust. This foundation enabled them to handle the challenges and responsibilities of their role more effectively, fostering a positive sense of independence. The respondents expressed that they received sufficient support, allowing them to navigate the ups and downs of their job. Collaboration with colleagues made work easier and provided them with the security and guidance they needed to act independently when required.

I am happy to work independently and that I want space to act and take action myself. But when I have the need and let it be known, then I want to get support. So, the trust thing is really important, I think, between, between a manager and their manager. [Authors note: referring to fellow first-line managers and their manager] (IP2)

The first-line managers also emphasized the importance of having an office on the unit, a reasonable workload with a suitable number of employees to work with, and access to necessary support functions to manage day-to-day operations effectively. They believed these were essential prerequisites to remain in the role and important for continuing their managerial assignments. The first-line managers expressed that it was important to have well-functioning support from the organization, especially regarding finances and human resources (HR), in addition to having access to close support functions. They believed that having such access was central to performing their work in the best possible way, as it eased their workload and helped them make the right decisions, which in turn contributed to their willingness to remain in managerial roles.

Well. . . it’s the support functions like finance, HR. . . managerial support; those are important, and that the people in those roles are professional (IP5)

### Maintaining Responsibility Through Boundary-Setting and Experience

The first-line managers perceived that setting boundaries and applying their accumulated experience were crucial for them to continue in their role, as these aspects helped them manage their work and leisure time effectively. They acknowledged their health and well-being as their own responsibility and saw boundary-setting as a means to prevent overworking and promote well-being, creating positive energy both at work and during leisure time. They believed it was crucial to avoid overly long working days, and, during particularly demanding periods, took time off to recover—a practice their manager often encouraged, recognizing that maintaining a healthy balance was essential to their long-term commitment to the managerial role.

*I* rarely work past 5 PM anymore, in the past my work hours often extended until *6* or 7 PM, and occasionally even as late as 8 PM, and then *I leave* the computer. Additionally, I now keep my weekends completely free of work. (IP20)

They emphasized that continuity in the role and their experience allowed them to understand and manage their unit better and prioritize tasks within the organization. This experience had also taught them how to balance work and leisure in a healthier way; they actively chose not to work on weekends and put down their phones to disconnect, which they felt was essential for recovery and a sound work-life balance. These practices contributed to their personal well-being, enhancing their effectiveness as managers and enabling them to lead their teams more efficiently. They believed that having a job with a clear purpose and defined boundaries increased their job satisfaction. Flexibility at work was also highly valued, particularly when managing family responsibilities.

If you compare how I managed my unit 11 years ago to how I do it now, you’ll see I’ve developed significantly. I’ve come to realize what really matters (IP 1)

### Making a Difference for Employees and Patients in a Collaborative and Forward-Thinking Unit

The first-line managers expressed a deep sense of joy and commitment in their work, which motivated them to remain in their role. They saw their relationship with employees as central to building a unit that met individual needs and fostered a sense of belonging and appreciation. Creating a workplace where both first-line managers and employees could thrive, feel valued, and enjoy their work was of utmost importance to them. They viewed their role as a meaningful opportunity for personal growth, allowing them to make a difference for both employees and patients. They described the pride they felt in their work, emphasizing how positively impacting the well-being of others added deep meaning to their role and inspired them to do their best, even in challenging situations. The first-line managers found motivation in being involved in positive changes and seeing the results of their hard work. They were driven to improve patient care and create a helpful environment. Engaging in strategic discussions and influencing the development of a forward-thinking unit was rewarding for them, as they valued the chance to contribute to an adaptable, future-focused unit.

I think it’s fun, what’s fun is being able to be part of the everyday life and development of the unit (IP8)

Establishing a stable and harmonious work environment was a priority, as was the satisfaction of solving complex challenges and contributing to projects that improved patient outcomes. They felt a sense of accomplishment when successfully addressing obstacles, which reinforced their pride in their work and role. Despite the challenges and occasional loneliness of the position, the first-line managers found their role both enriching and stimulating. They were proud of building a cohesive and resilient team and were motivated by the opportunity to continue developing both themselves and their team members. This ongoing growth fueled their commitment to the role, helping them maintain a long-term perspective and desire to stay in the managerial position, where they felt they could make a lasting, positive impact to the patients and employees at their unit.

So that, that is what drives me, that I want to make things better for the patients. And then I want it better for the employees, but if things are better for the patients, they’ll be better for the employees too. (IP 3)

## Discussion

This study highlights how first-line managers’ motivation to remain in their roles is shaped by supportive relationships, a sense of autonomy, and meaningful engagement in daily work. Our findings suggest that boundary-setting is not only a strategy to protect well-being, but also a way for managers to take ownership of their role in a sustainable way. Experiencing trust and encouragement build confidence and created a collaborative environment that helped them handle challenges effectively. Our findings reinforce earlier research showing that supportive infrastructures are important for first-line managers to sustain performance and remain committed.^11^ This is further supported by a repeated cross-sectional study by Svanström et al,^19^ which over 4 consecutive years demonstrated that high job demands and poor workplace relationships were negatively associated with job satisfaction. In contrast, perceived control over one’s work, support from managers and peers, role clarity, and participation in change processes were positively linked to satisfaction among first-line healthcare managers. These findings align with our participants’ emphasis on trust, access to support, and the ability to influence their work environment. Defining limits and drawing on experience enhanced their well-being and sustained their engagement. Flexibility in managing schedules supported a healthy balance between professional and personal responsibilities. The link between meaningfulness in the role and motivation to stay illustrates how personal values and organizational goals can align to foster long-term engagement. Opportunities for growth and contributing to positive outcomes at their unit further reinforced their commitment to the role.

First-line managers who perceived trust and support from their manager also had an increased capacity for autonomous decision-making, a factor they identified as critical for sustaining their roles within the organization. This aligns with findings from Conley,^25^ who emphasized the necessity of managerial autonomy for impactful decision-making among first-line managers. Similarly, Penconek et al,^11^ demonstrated that enhancing autonomy, fostering social support, and minimizing job-related stress significantly improve job satisfaction among first-line managers. Our findings underscore the role of a cohesive and well-coordinated management team in establishing a foundation of trust, thereby fostering a constructive work environment that energizes first-line managers and provides a sense of security crucial for their role commitment.

Furthermore, trust-building within managerial teams is widely recognized as a key driver not only for the motivation of first-line managers but also for the alignment of their goals with those of the organization as a whole.^26^ Our findings also reveal that collaborative practices among first-line managers are instrumental in reinforcing their role satisfaction. Within this collaborative framework, mutual respect enhances their confidence in executing responsibilities independently while promoting peer mentoring, constructive feedback, and collective resilience. These findings are consistent with Lundin et al,^18^ who observed that first-line managers value mutual support from peers, especially during periods of intensified workload. Such peer support has a dual impact: it bolsters productivity and aids retention by equipping managers with the resilience needed to navigate challenging periods together. Overall, our findings suggest that a well-functioning, collaborative environment not only enhances professional growth and resilience but also plays a pivotal role in the retention of first-line managers within healthcare organizations.

The participants in our study underscored the importance of having access to both close support functions and organizational support functions such as HR in their daily work. A recent review by Keith et al^9^ affirms that administrative support is vital in establishing a healthy work environment for first-line managers. Our findings indicate that reliable access to resources like HR and financial support helps first-line managers feel more secure and focused on their responsibilities. This support not only reduces stress but also strengthens their confidence in handling daily challenges, contributing to a sense of stability in their roles. While previous research has shown that administrative support improves job satisfaction and reduces stress,^11,27^ our findings further suggest that such support is also crucial for fostering resilience and encouraging first-line managers to remain in their roles. This perspective extends previous findings that first-line managers face high stress due to the dual demands of managing both organizational and administrative responsibilities.^28^ Furthermore, a lack of organizational assistance has been found to increase turnover among first-line managers.^9^ Similarly, a longitudinal cohort study by Svanström et al^29^ found that a lack of managerial support and limited control over one’s work were significant predictors of actual turnover among first-line managers, whereas factors such as team size or interpersonal tensions were not. This supports our findings that autonomy, trust, and accessible support systems are crucial for long-term retention in these roles. Thus, organizational support is essential for enhancing job satisfaction and may reduce turnover, thereby promoting continuity in healthcare management and aiding in the retention of first-line managers.

Boundary-setting was revealed as an essential strategy for first-line managers long-term commitment to the managerial role. In line with existing literature, which emphasizes the importance of managing boundaries to mitigate the risks associated with overwork in high-demand environments,^12,30,31^ our results offers new insights by demonstrating that boundary-setting is not only a supportive aspect but it might also be an intentional strategy that strengthens motivation and sustainability in the managerial role. This finding is especially relevant given the increasing turnover rates among healthcare managers, partly driven by challenges in maintaining a work-life balance,^28^ with one of the reasons being the imbalance between work and personal life experienced by first-line managers.

Accumulated experience allowed first-line managers to develop nuanced strategies for prioritizing tasks and setting boundaries, helping them sustain both personal well-being and operational efficiency. While previous research^8,11^ underscores the importance of experience in crafting effective work-life balance strategies, this study uniquely highlights how first-line managers’ accumulated experience empowers them to make strategic decisions that enhance both team management and workload prioritization. Flexibility in managing work boundaries was also essential for many managers, particularly in balancing family responsibilities. They prioritized health and well-being by setting boundaries, such as avoiding long workdays and taking necessary time off with support from their manager. This approach aligns with findings by Adriaenssens et al,^17^ which similarly emphasizes that supportive work conditions, including flexibility, enhance job satisfaction and retention. Additionally, first-line managers found that disconnecting on weekends and minimizing work-related stress during personal time were vital for recovery. These proactive strategies mirror findings by Warshawsky et al,^3^ who underscore that retaining committed managers positively impacts team morale and organizational stability. Our study contributes new insights by demonstrating that accumulated experience not only aids in boundary-setting but also strengthens resilience, promoting both individual well-being and a positive organizational environment in healthcare settings.

A notable finding was that first-line managers were motivated to remain in their roles when they had the opportunity to work closely with their employees and develop their units. While previous research highlights that conflicts with employees can sometimes drive first-line managers to leave their positions,^32^ our findings suggest that fostering collaborative relationships and focusing on unit development might increase the likelihood of managers staying in their roles. This result aligns with those of Lundin et al,^18^ who found that first-line managers underlined the importance of establishing positive and encouraging relationships with their employees as it provided a solid foundation for constructive assistance. By prioritizing teamwork and employee development, first-line managers can create a more positive work environment, leading to increased job satisfaction and a higher likelihood of staying in the managerial role. Additionally, Papadopoulos et al^33^ found that first-line managers who support their employees build stronger relationships characterized by trust, openness, and mutual respect. Such relationships not only foster collaboration and teamwork but also enhance communication and morale, contributing to a positive and productive workplace atmosphere. This aligns with our findings that first-line managers described a strong sense of pride in their work, driven by opportunities to develop their units and positively impact both employees and patients. This is reinforced by Penconek et al,^11^ who emphasized the importance of a developing organizational environment for retention. The result in our study showed that first-line managers valued contributing to patient care and team cohesion, which enhanced their job satisfaction and strengthened their commitment to remaining in their role.

### Strength and Limitations

This study provides essential insights into the organizational, role-related, and individual conditions influencing first-line managers’ decisions to remain in their positions. A key strength is its qualitative design, which captures first-line managers’ nuanced perspectives and experiences, often missed in quantitative studies. The purposive sampling approach, incorporating diverse healthcare sectors and first-line managers with varying responsibilities, enhances the study’s relevance and applicability. To achieve trustworthiness, the research process was guided by established criteria in qualitative research: credibility, dependability, confirmability, and transferability. For credibility, the alignment of data collection with the research objectives and the inclusion of varied participant experiences offered diverse viewpoints. Dependability was strengthened by iterative team discussions. Transferability was supported by detailed contextual descriptions and representative quotes. Additionally, the study adhered to the SRQR guidelines,^22^ ensuring transparency and reproducibility. Nevertheless, the study has limitations that should be acknowledged. First, the interviews were conducted by the first author, who had limited prior experience in qualitative research. Although preparatory steps were taken (eg, pilot interviews and supervision), this may initially have influenced interview dynamics or probing depth. Yet, the author’s interviewing skills and confidence developed throughout the data collection process, supported by ongoing reflection and guidance. That the interview guide was specifically developed for this study could be considered as a second limitation. However, it was pilot-tested to check clarity and relevance, and no major changes were needed. All interviews were conducted via Zoom, which, while practical, may have limited the ability to capture non-verbal cues or foster deeper rapport. Additionally, the timing of data collection in the immediate aftermath of the lifting of national restrictions in Sweden, allowed for post-restriction perspectives to be captured. However, pandemic-specific insights may limit applicability to routine conditions, they provide valuable strategies for supporting managers during both crises and more stable periods. Although the gender distribution among participants might seem imbalanced at first glance, with only 3 of the 20 first-line managers being men, this mirrors the actual distribution within the Swedish healthcare sector, where women constitute the majority at this leadership level. While the study was conducted in a specific region in Sweden, and the findings may not fully generalize to other settings, the identified theme and categories are likely to resonate broadly, with adaptations to local contexts.

### Implication for Practice

The findings from this study provide practical insights for healthcare organizations seeking to retain first-line managers. Establishing trust through supportive and transparent leadership can enhance job satisfaction and resilience among managers. Organizations should also focus on maintaining manageable workloads, to help alleviate stress and ensure a healthy work-life balance. Creating a work environment that emphasizes meaningful contributions to patient care and team development is likely to improve overall satisfaction and retention. Healthcare organizations may consider steps such as introducing mentorship programs, offering tailored professional development opportunities, and enacting policies that promote work-life balance. Encouraging collaboration among management teams can further strengthen resilience and support. By addressing these aspects organizations can create a more sustainable and supportive environment for first-line managers, contributing to organizational stability and quality healthcare delivery. Importantly, the core components identified in this study; trust, autonomy, peer support, and boundary-setting are not only vital for first-line managers but may also positively influence other levels of management. Strengthening these aspects at the base of the leadership structure can contribute to a more cohesive, resilient, and sustainable leadership culture throughout the healthcare organization.

## Conclusion

Trust from their manager, autonomy in decision-making, and support from colleagues enhance first-line managers’ sense of purpose and motivation, encouraging them to remain in their roles. Moreover, balanced workloads and opportunities to make a meaningful impact strengthen their commitment. By recognizing and addressing these aspects, healthcare organizations can better support the long-term engagement of first-line managers, fostering sustainable leadership, and contributing to greater stability in healthcare organizations.

## Supplemental Material

sj-doc-2-inq-10.1177_00469580251368724 – Supplemental material for “Trust, Responsibility, and Making a Difference”: Qualitative Insights into Why First-Line Managers in Healthcare Remain in Their Role Supplemental material, sj-doc-2-inq-10.1177_00469580251368724 for “Trust, Responsibility, and Making a Difference”: Qualitative Insights into Why First-Line Managers in Healthcare Remain in Their Role by Jonas Svanström, Magnus Lindberg, Bernice Skytt and Maria Lindberg in INQUIRY: The Journal of Health Care Organization, Provision, and Financing

sj-docx-1-inq-10.1177_00469580251368724 – Supplemental material for “Trust, Responsibility, and Making a Difference”: Qualitative Insights into Why First-Line Managers in Healthcare Remain in Their Role Supplemental material, sj-docx-1-inq-10.1177_00469580251368724 for “Trust, Responsibility, and Making a Difference”: Qualitative Insights into Why First-Line Managers in Healthcare Remain in Their Role by Jonas Svanström, Magnus Lindberg, Bernice Skytt and Maria Lindberg in INQUIRY: The Journal of Health Care Organization, Provision, and Financing
