# Supplementary material for: “Trust, Responsibility, and Making a Difference”: Qualitative Insights into Why First-Line Managers in Healthcare Remain in Their Role
Source: Inquiry. 2025 Aug 21;62:00469580251368724. doi: 10.1177/00469580251368724 (PMC12374037; doi:10.1177/00469580251368724)
Supplement: sj-docx-1-inq-10.1177_00469580251368724 – Supplemental material for “Trust, Responsibility, and Making a Difference”: Qualitative Insights into Why First-Line Managers in Healthcare Remain in Their Role [file sj-docx-1-inq-10.1177_00469580251368724.docx]

**Interview guide**

The aim of this study is to describe first-line managers' perceptions of what is important for them to remain in the managerial role.

Sex:______

Age:______

Education (graduation year):_____________________________

Total years of experience working as a manager:______

Which prerequisites are important for you in your work in order for you to continue your managerial role?

What motivates you to continue your mission as a first-line manager? (e.g. Personal reasons, organizational reasons, management)

How do you see your work in the coming years?
